# Role of Phenylalanine and Valine^10^ Residues in the Antimicrobial Activity and Cytotoxicity of Piscidin-1

**DOI:** 10.1371/journal.pone.0114453

**Published:** 2014-12-04

**Authors:** Eunjung Lee, Areum Shin, Ki-Woong Jeong, Bongwhan Jin, Hum Nath Jnawali, Soyoung Shin, Song Yub Shin, Yangmee Kim

**Affiliations:** 1 Department of Bioscience and Biotechnology, Bio-Molecular Informatics Center, Konkuk University, Seoul, Korea; 2 Research Center for Proteineous Materials and Department of Cellular & Molecular Medicine, School of Medicine, Chosun University, Gwangju, Korea; Swinburne University of Technology, Australia

## Abstract

Piscidin-1 (Pis-1) is a linear antibacterial peptide derived from mast cells of aquacultured hybrid striped bass that comprises 22 amino acids with a phenylalanine-rich amino-terminus. Pis-1 exhibits potent antibacterial activity against pathogens but is not selective for distinguishing between bacterial and mammalian cells. To determine the key residues for its antibacterial activity and those for its cytotoxicity, we investigated the role of each Phe residue near the N-terminus as well as the Val^10^ residue located near the boundary of the hydrophobic and hydrophilic sectors of the helical wheel diagram. Fluorescence dye leakage and tryptophan fluorescence experiments were used to study peptide-lipid interactions, showing comparable depths of insertion of substituted peptides in different membranes. Phe^2^ was found to be the most deeply inserted phenylalanine in both bacterial- and mammalian-mimic membranes. Each Phe was substituted with Ala or Lys to investigate its functional role. Phe^2^ plays key roles in the cytotoxicity as well as the antibacterial activities of Pis-1, and Phe^6^ is essential for the antibacterial activities of Pis-1. We also designed and synthesized a piscidin analog, Pis-V10K, in which Lys was substituted for Val^10^, resulting in an elevated amphipathic α-helical structure. Pis-V10K showed similar antibacterial activity (average minimum inhibitory concentration (MIC)  = 1.6 µM) to Pis-1 (average MIC  = 1.5 µM). However, it exhibited much lower cytotoxicity than Pis-1. Lys^10^-substituted analogs, Pis-F1K/V10K, Pis-F2K/V10K, and Pis-F6K/V10K in which Lys was substituted for Phe retained antibacterial activity toward standard and drug-resistant bacterial strains with novel bacterial cell selectivity. They exert anti-inflammatory activities via inhibition of nitric oxide production, TNF-α secretion, and MIP-1 and MIP-2 production. They may disrupt the binding of LPS to toll-like receptors, eventually suppressing MAPKs-mediated signaling pathways. These peptides may be good candidates for the development of peptide antibiotics with potent antibacterial activity but without cytotoxicity.

## Introduction

In recent years, a rapid increase in the emergence of microbes that are resistant to conventionally used antibiotics has been observed [1]. To treat infections caused by multidrug-resistant bacteria, the inevitable search for new antibiotics has become urgent. Among the possible candidates, antimicrobial peptides have attracted increased clinical interest, followed by expanded research. Antimicrobial peptides, which have been isolated from various origins, including animals, plants, insects, amphibians, and bacteria [2], provide immediate protection by exerting a direct physicochemical attack on the surface membranes of microorganisms. The antimicrobial activities of antimicrobial peptides play important roles in the host defense system, and the innate immunity of all species is related to the ability of the peptides to adopt an amphipathic structure, including an α-helix, a β-sheet, or aβ-turn. Although the detailed mechanisms have not been fully elucidated, the antibiotic action of antimicrobial peptides appears to involve depolarization or permeabilization of the bacterial cell membrane [3]–[7].

Piscidins were the first family of antimicrobial amphipathic cationic peptides to be isolated from the mast cells of aquacultured hybrid striped bass (*Morone chrysops* × *M. saxatilis*). Mast cells, which are immune cells of uncertain function, are present in all vertebrates [8]–[10]. Piscidin plays important roles in the innate immune system of fish, and, of all the piscidin peptides identified to date, piscidin 1 (Pis-1) shows the highest antibacterial activity [8]–[10]. Pis-1 exhibits potent activity against a wide variety of pathogens such as fungi, yeast, as well as Gram-positive and -negative bacteria (including antibiotic-resistant bacteria). However, the therapeutic usefulness of this peptide may be limited because it causes hemolysis and cytotoxicity. According to the findings of our previous study, Pis-1 has an α-helical structure from Phe^2^ to Thr^21^ in sodium dodecyl sulfate micelles and is not selective against bacterial versus mammalian cells. However, Pis-1 [PG], in which Pro has been substituted for Gly^8^, and Pis-1 [NkG], in which a Lys peptoid residue has been substituted for Gly^8^, have a hinge structure in between the two helices and show higher bacterial cell selectivity than Pis-1 [8]–[14]. The active antiparasitic effects of piscidin 2 (Pis-2) against the infective stage of parasites suggested that Pis-2 and its correlated peptides are potent components of antimicrobial defense in various fish species [15]. Piscidin 3 (Pis-3) showed lower hemolytic activity than Pis-1or melittin, a peptide derived from bee venom. In Pis-3, a glycine is substituted for the histidine at position 17 in Pis-1, and Pis-3 tends to show lower amphipathicity. This structural distinction between Pis-1 and Pis-3 is related to the reduced hemolysis [9]. Piscidin 4 (Pis-4), another antimicrobial peptide isolated from mast cells of hybrid striped bass, comprises 44 amino acid residues and exhibits distinct N-terminal sequence homology with Pis-1, Pis-2, and Pis-3. The N-terminal residues (amino acids 1-11) form an α-helix and C-terminal residues (amino acids 33–44) form a random coil with highly acidic residues in Pis-4. Pis-4 shows lower α-helicity than Pis-1 in hydrophobic environments since Pis-4 includes more Gly and Pro which cause a flexibility and bend. Pis-4 exhibites less hemolytic activirty than Pis-1, implying that C-terminal random coil region of Pis-4 prevents the insertion into hydrophobic erythrocyte membrane. On the other hand, Pis-4 shows very high activity toward DPPC and EYPC liposome despite Pis-4 containing very low α-helical contents. This finding indicates that α-helix is not solely responsible for its selectivity [16]. Therefore, it is necessary to verify the key residues that are important for the cytotoxicity as well as antibacterial activities of piscidins. In this study, we confirmed and investigated the roles of key residues such as phenylalanines and Valine^10^ that are most important for the cytotoxicity and antibacterial activity of Pis-1, the piscidin with strongest antibacterial activity. It is well known that hydrophobicity influences not only the antimicrobial activity but also the cytotoxicity of antimicrobial peptides [17].

In this study, to investigate the membrane position and functional role of each Phe residue in Pis-1, the blue-shift of the tryptophan fluorescence ofthe Trp-substituted peptides of Pis-1 was monitored. In Pis-F1A, Pis-F2A, and Pis-F6A, each Phe was substituted with an Ala to investigate their functional roles. Phe was also substituted with Lys to examine the influence of increasing the positive charge of the molecule. In order to enhance the bacterial cell selectivity, we then designed Pis-V10K, in which Lys was substituted for Val^10^ located near the boundary of the hydrophobic and hydrophilic sector of the helical wheel diagram. This produced an enhanced amphipathic α-helical structure, which was utilized as the template for all analogs. Phe^1^, Phe^2^, and Phe^6^ were further substituted with Lys to produce Pis-F1K/V10K, Pis-F2K/V10K, and Pis-F6K/V10K, respectively, to increase cationicity and decrease hydrophobicity. We evaluated the toxicities of these analogs toward bacterial and mammalian cells, as well as their ability to permeabilize model phospholipid membranes. Elucidating the structure-antibiotic activity relationships of these peptides could help clarify their selectivities for bacterial cell membranes, and thus aid in the designing of potent bacterial-selective antimicrobial peptides.

## Materials and Methods

### Peptide Synthesis

All peptides specified in Table 1 were prepared by solid-phase synthesis using *N*-(9-fluorenyl) methoxycarbonyl (Fmoc) chemistry. Peptides were purified by reversed-phase preparative high-performance liquid chromatography on a C_18_ column (20×250 mm; Shim-pack) using a 10–80% H_2_O/acetonitrile gradient with 0.1% trifluoroacetic acid delivered over 30 min. Analytical high-performance liquid chromatography with an ODS column (4.6×250 mm; Shim-pack) revealed that purified peptides were more than 95% homogeneous (data not shown). The peptides also had the correct atomic masses, as determined by matrix-assisted laser desorption/ionization time-of-flight mass spectrometry (MALDI-TOF MS) (Shimadzu; Kyoto, Japan). Peptide concentrations were quantified using bradford assay by Cary 50 UV-Vis spectrophotometer (Varian Inc., Santa Clara, CA, USA).

**Table 1:** Amino acid sequences and molecular mass data for Pis-1 and its analogs.

Peptide	Sequence	Molecular mass (Da)	Net charge^a^	Mean hydrophilicity^b^
Pis-1 (native)	FFHHIFRGIVHVGKTIHRLVTG	2572.1	3.4	−0.59
Pis-F1A	**A**FHHIFRGIVHVGKTIHRLVTG	2496.0	3.4	−0.50
Pis-F2A	F**A**HHIFRGIVHVGKTIHRLVTG	2496.0	3.4	−0.50
Pis-F6A	FFHHI**A**RGIVHVGKTIHRLVTG	2496.0	3.4	−0.50
Pis-F1K	**K**FHHIFRGIVHVGKTIHRLVTG	2553.1	4.4	−0.34
Pis-F2K	F**K**HHIFRGIVHVGKTIHRLVTG	2553.1	4.4	−0.34
Pis-F6K	FFHHI**K**RGIVHVGKTIHRLVTG	2553.1	4.4	−0.34
Pis-F1W	**W**FHHIFRGIVHVGKTIHRLVTG	2611.1	3.4	−0.63
Pis-F2W	F**W**HHIFRGIVHVGKTIHRLVTG	2611.1	3.4	−0.63
Pis-F6W	FFHHI**W**RGIVHVGKTIHRLVTG	2611.1	3.4	−0.63
Pis-V10K	FFHHIFRGI**K**HVGKTIHRLVTG	2601.1	4.4	−0.39
Pis-F1K/V10K	**K**FHHIFRGI**K**HVGKTIHRLVTG	2582.1	5.4	−0.14
Pis-F2K/V10K	F**K**HHIFRGI**K**HVGKTIHRLVTG	2582.1	5.4	−0.14
Pis-F6K/V10K	FFHHI**K**RGI**K**HVGKTIHRLVTG	2582.1	5.4	−0.14

a Net charge was calculated using the sum of each of the amino acid charges at pH 7.0 [36].

b The mean hydrophilicity(H), is the total hydrophilicity (sum of all residue hydrophilicity indices) divided by the number of residues according to the Hopp and Woods scale [37].

### Antibacterial Activity

*Escherichia coli* KCTC 1682, *E. coli* KCTC 2593, *E. coli* KCTC 2571, *Salmonella typhimurium* KCTC 1926, *Pseudomonas aeruginosa* KCTC 1637, *P. aeruginosa* KCTC 2004, *P. aeruginosa* KCTC 2513, *Bacillus subtilis* KCTC 3068, *B. subtilis* KCTC 1021, *B. subtilis* KCTC 1022, *Staphylococcus epidermidis* KCTC 1917, *Staphylococcus aureus* KCTC 1621, *S. aureus* KCTC 1916, and *S. aureus* KCTC 3881 were purchased from the Korean Collection for Type Cultures, Korea Research Institute of Bioscience & Biotechnology (Taejon, Korea). Methicillin-resistant *S. aureus* (MRSA) (CCARM 3089, CCARM 3090, CCARM 3108, CCARM 3114, and CCARM 3126), multidrug-resistant *E. coli* (MDREC) (CCARM 1229 and CCARM 1238), multidrug-resistant *S. typhimurium* (MDRST) (CCARM 8003, CCARM 8007, and CCARM 8009), multidrug-resistant *Acinetobacter baumanii* (MDRAB) (CCARM 12005, CCARM 12035, CCARM 12036, CCARM 12037), and multidrug-resistant *P. aeruginosa* (MDRPA) (CCARM 2002, CCARM 2003, CCARM 2095, and CCARM 2163) were obtained from the Culture Collection of Antibiotic-Resistant Microbes (CCARM) at Seoul Women’s University in Korea. The antimicrobial activities of the peptides against selected organisms, including three Gram-positive, three Gram-negative, and five antibiotic-resistant bacteria, were determined using a broth microdilution assay. Briefly, single colonies of bacteria and fungi were inoculated into Luria-Bertani (LB) medium and cultured overnight at 37°C. An aliquot of the culture was transferred to 10 mL of fresh LB and incubated for an additional 3–5 h at 37°C until it reached the mid-logarithmic phase. Two-fold serial dilutions of the peptides in 1% peptone were prepared. The serial dilutions (100 µL) were added to 100 µL of cells (2×10^6^ colony-forming units (CFU)/mL) in 96-well microtiter plates and incubated at 37°C for 16 h. The lowest concentration of peptide that completely inhibited growth was defined as the minimal inhibitory concentration (MIC). MIC values were calculated as the average of triplicate measurements from three independent assays.

### Ethics Statement

These procedures were approved by the Institutional Review Board of Konkuk University, and all donorsprovided written informed consent before the experiments were conducted. We followed the Ethical Standards of the Institutional Review Board of Konkuk University. Human red blood cells (hRBCs) were obtained from healthy volunteer donors at the Konkuk University Hospital in Seoul (Republic of Korea). This study received ethical approval from the Institutional Review Board of Konkuk University. The relevant information for blood donors, except age and gender, was provided by an anonymous source. Blood samples were provided by 3 healthy volunteer donors. The samples were immediately blinded for use in the study and unused samples were immediately discarded.

### Hemolysis

The hemolytic ability of the peptides was tested using hRBCs. FreshhRBCs were washed three times with phosphate-buffered saline (PBS) (35 mM phosphate buffer containing 150 mM NaCl, pH 7.4), centrifuged for 10 min at 1000×*g*, and resuspended in PBS. The peptide solutions were then added to 50 µL of hRBCs in PBS to yield a final volume of 100 µL and a final erythrocyte concentration of 4% (v/v). The resulting suspension was incubated with agitation for 1 h at 37°C. The samples were centrifuged at 1000×*g* for 5 min. Hemoglobin release was monitored by measuring the absorbance of the supernatant at 405 nm. Controls for zero hemolysis (blank) and 100% hemolysis consisted of hRBCs suspended in PBS and 0.1% Triton-X 100, respectively. The percentage of hemolysis was calculated using the following equation:
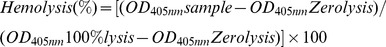



### Cytotoxicity

The mouse embryonic fibroblast-derived cell line NIH3T3 was purchased from the American Type Culture Collection (Manassas, VA). Cells were cultured in Dulbecco’s modified Eagle medium (DMEM) supplemented with 10% fetal bovine serum (FBS) and antibiotic solution (100 units/mL penicillin and 100 µg/mL streptomycin) at 37°C in a humidified 5% CO_2_ atmosphere. Cultures were passaged every 2–3 days by brief trypsin treatment and visualized with an inverted microscope. The cytotoxicity of peptides against mammalian cells was determined using a 3-(4,5-dimethylthiazol-2-yl)-2,5-diphynyltetrazolium bromide (MTT) assay. Cells were seeded on 96-well microplates at a density of 1×10^4^ cells/well in 100 µL of DMEM containing 10% FBS and 1% antibiotics, and NIH3T3 cells were seeded on 96-well microplates at a density of 1×10^4^ cells/well in 100 µL of DMEM containing 10% FBS and 1% antibiotics. Plates were incubated for 24 h at 37°C in 5% CO_2_. Serial 2-fold dilutions of peptide solutions (100 µL) in DMEM were added; wells containing cells without peptides served as controls. After incubating plates for 1 d, 20 µL of MTT solution (5 mg/mL) was added to each well, and the plates were incubated for an additional 4 h at 37°C. Precipitated MTT formazan was dissolved by adding 100 µL of dimethyl sulfoxide. Absorbance at 570 nm was measured using an enzyme-linked immunosorbent assay (ELISA) reader (Molecular Devices; Sunnyvale, CA). Cell survival, expressed as a percentage, was calculated as the ratio of A_570_ for cells treated with peptide to that of cells without peptide treatment.

### CD Analysis

CD experiments were performed using a J810 spectropolarimeter (Jasco, Tokyo, Japan) with a 1-mm path-length cell. The CD spectra were recorded at 25°C at 0.1-nm intervals from 190 to 250 nm. To investigate the conformational changes induced by membrane environments, 50 mM dodecylphosphocholine (DPC) micelles,100 mM sodium dodecyl sulfate (SDS) micelles, 1 mg/mL LPS, and 1 mM solutions of SUVs comprising egg yolk PC/egg yolk PG (7∶3, w/w) with a defined composition were added to the peptide solution. Peptide concentration of CD measurements was 50 µM. For each spectrum, the data from 10 scans was averaged and smoothed using J-810 spectrometer. CD data were expressed as the mean residue ellipticity [θ] in deg⋅cm^2^⋅dmol^−1^. The percentage of α-helical structure was calculated as follows:
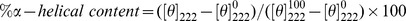



where [*θ*]_222_ is the experimentally observed mean residue ellipticity at 222 nm, and values for [*θ*]^0^_222_ and [*θ*]^100^_222_, which correspond to 0% and 100% helix content at 222 nm, are estimated to be −2000 and −30000, respectively [18].

### Tryptophan Fluorescence Experiments

Small unilammellar vesicles (SUVs) were prepared according to a standard procedure using the required amounts of egg yolk L-α-phosphatidylcholine (EYPC)/egg yolk L-α-phosphatidylglycerol (EYPG) (7∶3, w/w), and EYPC/cholesterol (CH) (10∶1, w/w). Dry lipids were dissolved in chloroform, deposited as a film on the wall of a glass vessel, and then lyophilized overnight. Dried thin films were resuspended in Tris-HCl buffer by vortexing. The lipid dispersions were then sonicated in an ice-water bath for 10–30 min using a titanium-tipped ultrasonicator until the suspension become transparent. SUVs were added to a peptide solution (5 µM concentration) in 10 mM Tris buffer, pH 7.4, 0.1 mM ethylenediaminetetraacetic acid (EDTA), and 150 mM NaCl and maintained at 25°C with continuous stirring in a total volume of 2 mL. The lipid concentration of the SUVs was 1 mM. Tryptophan residues of each peptide were excited at 280 nm, and emission spectra were recorded in the 300–400 nm range, using a 5-nm bandpass filter. Blue shifts were also measured in 8.0 µM lipopolysaccharide (LPS) and 1.5 mM dodecylphosphocholinein 10 mM sodium phosphate buffer.

### Calcein Leakage Assay

Calcein-entrapped large unilamellar vesicles (LUVs) composed of EYPC/EYPG (7∶3, w/w)and EYPC/CH (10∶1, w/w) were prepared by vortexing dried lipids in dye buffer solution (70 mM calcein, 10 mM Tris, 150 mM NaCl, 0.1 mM EDTA, pH 7.4). The suspension was subjected to ten freeze-thaw cycles in liquid nitrogen and extruded through polycarbonate filters (two stacked 100-nm pore-size filters) using a LiposoFast extruder (Avestin, Inc.; Ottawa, Canada). Untrapped calcein was removed by gel filtration on a Sephadex G-50 column. Passing through a Sephadex G-50 column usually resulted in an approximately 10-fold dilution of lipid vesicles. The eluted calcein-entrapped vesicles were diluted further to achieve the desired final lipid concentration of 64 µM for the experiments. Leakage of calcein from LUVs was monitored by measuring fluorescence intensity at an excitation wavelength of 490 nm and an emission wavelength of 520 nm on a model RF-5301PC spectrophotometer (Shimadzu; Kyoto, Japan). Vesicles dissolved in 10% Triton X-100 in Tris-buffer (20 µL) were used to establish 100% dye-release and the total volume of the assay was 2 mL. The percentage of dye-leakage caused by the peptides was calculated as follows:
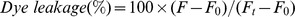



where F is the fluorescence intensity of peptide-treated vesicles, and *F*_0_ and *F*_t_ are the fluorescence intensities without peptides and with Triton X-100, respectively.

### Quantification of NO Production in LPS-stimulated RAW264.7 Cells

Nitrite accumulation in culture media was used as an indicator of NO production [19]. RAW264.7 cells were plated at a density of 1×10^5^ cells/mL in 96-well culture plates and stimulated with LPS (20 ng/mL) from *E. coli* O111∶B4 (Sigma) in the presence or absence of peptides for 24 h. Isolated supernatant fractions were mixed with an equal volume of Griess reagent (1% sulfanilamide, 0.1% naphthylethylenediamine dihydrochloride, 2% phosphoric acid) and incubated at room temperature (RT) for 10 min. Nitrite production was determined by measuring the absorbance at 540 nm, and converting values to nitrite concentrations using a standard curve generated with NaNO_2_.

### Quantification of Production of Inflammatory Cytokines in LPS-stimulated RAW264.7 Cells by ELISA

Antibodies against mouse tumor necrosis factor-α (mTNF-α) and mouse macrophage inflammatory protein 2 (mMIP-2) were immobilized on immunoplates by incubation with 0.2–0.8 µg/mL solutions of antibody in PBS overnight at RT. Plates were washed once with PBS/0.1% Tween-20 (PBST) and blocked by incubating with 200 µL of blocking solution (3% bovine serum albumin [BSA], 0.02% NaN_3_ in PBS) overnight at RT. Then, supernatants from LPS-stimulated RAW264.7 cells co-incubated with serially diluted peptide for 18 h were added to the wells of pre-coated plates and incubated for 2 h at RT. After washing the plates 3 times with PBST, biotinylated-anti-mTNF-α antibody (0.4 µg/mL) was diluted in 0.1% BSA and added, and plates were incubated for 2 h. Plates were then washed 3 times with PBST, and further incubated with streptavidin peroxidase (0.3 µg/mL) diluted in PBS. After washing, SureBlue 3,3′,5,5′-tetramethylbenzidine peroxidase substrate (KPL, Inc.; Gaithersburg, MD) was added. The enzyme reaction was allowed to proceed at RT for color development, and was stopped by adding 100 µL of 1 M H_2_SO_4_. The absorbance at 450 nm was measured using a microplate reader. All values represent the means ± standard deviations of at least 3 independent experiments [20].

### Reverse Transcription-Polymerase Chain Reaction (RT-PCR)

Because Pis-V10K and Pis-F2K/V10K showed inhibitory activities against NO, mTNF-α, and mMIP-2 production, we further investigated the inflammatory response pathway of Pis-V10K and Pis-F2K/V10K using RT-PCR. Mouse RAW264.7 cells were plated in 6-well plates (5×10^5^ cells/well) and cultured overnight. Cells were stimulated without (negative control) or with 20 ng/mL LPS in the presence or absence of peptide in RPMI-1640 supplemented with 1% penicillin/streptomycin for 3 h. After stimulation, cells were detached from the wells using cold PBS and washed once with PBS. Competitive RT-PCR was performed as previously described, with minor modifications [21]. Briefly, total RNA was extracted using an RNeasy kit (Qiagen;Hilden, Germany), according to the manufacturer’s instructions, and equal amounts of total RNA were reverse transcribed into cDNA using oligo(dT)-15 primers. The indicated targets were amplified from the resulting cDNA by PCR using the following specific primers: TNF-α, 5′-GTT CTG TCC CTT TCA CTC ACT G-3′ (sense) and 5′-GGT AGA GAA TGG ATG AAC ACC-3′ (antisense); MIP-1, 5′-ATG AAG CTC TGC GTG TCT GC-3′ (sense) and 5′-TGA GGA GCA AGG ACG CTT CT-3′ (antisense); MIP-2, 5′-ACA CTT CAG CCT AGC GCC AT-3′ (sense) and 5′-CAG GTC AGT TAG CCT TGC CT-3′ (antisense); and IL-1β,5′-CTG TCC TGA TGA GAG CAT CC-3′ (sense) and 5′-TGT CCA TTG AGG TGG AGA GC-3′ (antisense). The primers for glyceraldehyde-3-phosphate dehydrogenase (GAPDH), used as an internal control, were 5′-ACC ACA GTC CAT GCC ATC AC-3′ (sense) and 5′-TCC ACC ACC CTG TTG CTG TA-3′ (antisense). PCR was performed using the following cycling conditions: 94°C for 5 min, followed by 25 cycles of 94°C for 1 min, 55°C for 1.5 min, and 72°C for 1 min, and a final extension step of 72°C for 5 min. Amplified products were electrophoresed on 1% agarose gels, and bands were visualized by UV illumination of ethidium bromide-stained gels [21].

### Western blot

Raw264.7 cells were seeded in 6-well plates (3×10^6^ cells/well) and incubated in RPMI-1640 (Welgene, Daegu, Korea) supplemented with 10% FBS (Invitrogen, NY, USA) and antibiotics (100 U/mL penicillin and 100 µg/mL streptomycin; Invitrogen) at 37°C under 5% CO_2_. Cultured cells were stimulated with 20 ng/mL LPS for 3 h, after which cells were incubated overnight with peptides (25 µM). After incubation, cells were washed twice with PBS and detached with ice-cold PBS. The collected cells were centrifuged at 1000 rpm for 5 min at 4°C. Cell pellets were re-suspended in 100 µL of lysis buffer (1% Triton X-100, 1% deoxycholate, 0.1% NaN_3_) and incubated for 30 min on ice. Lysed cells were centrifuged at 12000 rpm for 10 min at 4°C, and the concentration of protein in the supernatant (cytoplasmic extract) was determined by Bradford assay (Bio-Rad, Hemel Hempstead, UK). Equal amounts of protein (20 µg) were separated by SDS-polyacrylamide gel electrophoresis (SDS-PAGE) on a 10% gel and transferred onto a polyvinylidene fluoride (PVDF) microporous membrane (Millipore, Billerica, MA, USA). The membrane was blocked by incubating with 5% bovine serum albumin (BSA) in TBST (25 mM Tris, 3 mM KCl, 140 mM NaCl, 0.1% Tween 20) for 1 h at RT, and then incubated with antibodies specific for toll-like receptor 2 (TLR2, 1∶2000; Cell Signaling Technology, Beverly, MA, USA), TLR4 (1∶2000; Cell Signaling Technology), phospho-p38 (1∶2000; Cell signaling Technology), phospho-ERK (1∶2000; Cell signaling Technology), phospho-JNK (1∶2000; Cell signaling Technology), and β-actin (1∶5000, Sigma-Aldrich, St. Louis, MO, USA). After washing with TBST buffer, the membrane was incubated with horseradish peroxidase-conjugated anti-rabbit IgG or anti-mouse IgG (1∶30000; Sigma-Aldrich, St. Louis, Missouri, USA) secondary antibodies, as appropriate. Signals were detected using an enhanced chemiluminescence (ECL) detection system (GE Healthcare, Buckinghamshire, UK).

### Fluorescein isothiocyanate (FITC)-labeled LPS Aggregates

The interaction of peptides with FITC-conjugated LPS was studied by exciting 0.5 µg/mL FITC-LPS at 480 nm and monitoring the change in the emission of FITC at 515 nm in the presence of different concentrations of peptide (0.1, 0.2, 0.4, 0.8, 1.6, 3.2, 6.4, 8.0, and 9.6 µM). Samples were prepared in 10 mM sodium phosphate buffer at pH 6.0 [22].

### Saturation transfer difference (STD) NMR experiments

Peptide (0.5 mM) was added to 0.1 mg/ml LPS in H_2_O at pH 5.9. STD-NMR experiments were recorded on a Bruker 500 MHz spectrometer at a temperature of 298 K. The STD-NMR spectra were obtained with 512 scans and selective saturation of LPS resonances at -2.0 ppm (40 ppm for reference spectra). A cascade of 40 selective Gaussian-shaped pulses of 45-ms duration and a 100-µs delay between each pulse were used in all STD-NMR experiments with a total saturation time of 2 s. Subtraction of the two spectra (on resonance - off resonance) leads to the difference spectrum, which contains signals arising from the saturation transfer. Therefore, spectral differences primarily constituted resonances belonging to peptide protons bound to LPS micelles.

## Results and Discussion

### Peptide Design

To investigate the roles of Phe residues in Pis-1 activity, the interaction of each Phe residue with the membranes was monitored using tryptophan fluorescence experiments, with analogs in which thePhe^1^, Phe^2^, and Phe^6^ residues had been replaced with Trp. To identify the contribution of the Phe residues to the activity of Pis-1, each Phe residue was substituted with Ala to produce Pis-F1A, Pis-F2A, and Pis-F6A. Ala is commonly used for examination of functional rolesof amino acidsin peptides or proteins because it is non-bulky and does not affect the secondary structure of the peptide or protein being investigated [23], [24]. Cationicity is an integral component of the interaction of antimicrobial peptides with the negatively charged phospholipid membranes of bacteria and other microorganisms. To determine how charge influences the activity of Pis-1, the Phe^1^, Phe^2^, and Phe^6^ residues were substituted with Lys, which is a positively charged residue.

**Figure 1:**
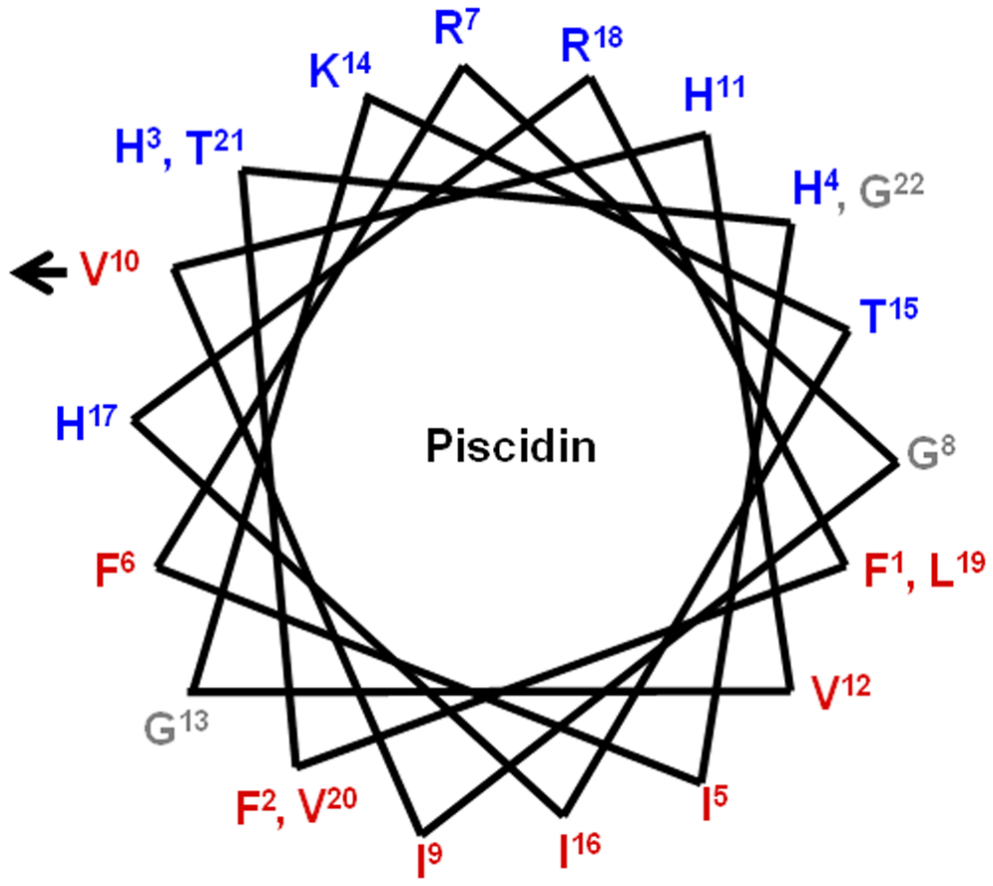
The α-helical wheel diagram for Pis-1. The arrows indicate the position of Val^10^ substituted with Lys. The Pis-1 wheel is amphipathic, with the hydrophobic residues in the lower part and the hydrophilic residues in the upper part.

Our previous study showed that Pis-1 exhibited high antibacterial activity, and it was also strongly cytotoxic. The helical wheel diagram of Pis-1 shown in Fig. 1 shows the amphipathicity of Pis-1, with the hydrophobic residues in the lower region and the hydrophilic residues in the upper region. As showin in Fig. 1, Pis-1 is highly cytotoxic because the hydrophobic sector is relatively large compared to the hydrophilic sector. Hydrophobicity, which influences not only the antimicrobial activity but also the cytotoxicity of antimicrobial peptides, can be reduced by replacing hydrophobic residues such as Phe or Val with positively charged residues such as Lys. Since Val^10^ located near the boundary between the hydrophobic and hydrophilic sector of the helical wheel diagram, substitution of Val^10^ with Lys would increase the size of hydrophilic sector and amphipathicity, resulting in decrease of cytotoxicity of Pis-1. Pis-V10K, in which Lys was substituted for Val^10^, should show higher amphipathicity than Pis-1. The sequence, net charge, and hydrophilicity of these peptides are listed in Table 1. To increase cationicity and decrease hydrophobicity, Phe^1^, Phe^2^, and Phe^6^ were further substituted with Lys to produce Pis-F1K/V10K, Pis-F2K/V10K, and Pis-F6K/V10K, respectively.

### Antibacterial Activities of the Peptides

The antibacterial activities of the peptides were examined against a representative set of standard bacterial strains, including 7 Gram-negative strains (*E. coli, S. typhimurium*, and *P. aeruginosa*) and 7 Gram-positive strains (*B. subtilis*, *S. epidermidis*, and *S. aureus*). We also measured the peptides’ antibacterial activities against 18 antibiotic-resistant bacterial strains (MRSA, MDREC, MDRST, MDRAB, and MDRPA). All peptides showed strong antibacterial activity not only against the standard bacterial strains but also against the antibiotic-resistant bacterial strains, as shown in Tables 2 and 3. Among the peptide analogs with substitution of Ala or Lys for Phe, Pis-F1A and Pis-F1K showed higher antibacterial activity than the Phe^2^- or Phe^6^-substituted Pis-1 analogs against both standard bacterial strains and drug-resistant bacterial strains. This indicated that Phe^1^, located at the N-terminus of Pis-1, does not play a critical role in its antibacterial activity. However, Pis-F6A and Pis-F6K showed 1.1–2.8-fold lower antibacterial activity against all bacterial strains than Phe^1^- or Phe^2^-substituted Pis-1 analogs. This finding indicates that Phe^6^ is important for the antimicrobial activity of Pis-1, and should therefore be retained in any designed peptide antibiotics. Trp-substituted mutants at Phe positions showed high antibacterial activities toward standard bacterial strains similar compared to Pis-1 and its analogs (Table S1). Even though blue shift data cannot be directly correlated with the depth of insertion of the peptides, these experiments may provide some hints about the depth of insertion of the peptides.

**Table 2:** Antimicrobial activity of Pis-1 and its analogs against standard bacterial strains.

	MIC^a^ (µM)										
Standard bacterial strains	Pis-1	Pis-F1A	Pis-F2A	Pis-F6A	Pis-F1K	Pis-F2K	Pis-F6K	Pis-V10K	Pis-F1K/V10K	Pis-F2K/V10K	Pis-F6K/V10K
*E. coli* KCTC1682	1.0	2.0	2.0	2.0	4.0	4.0	4.0	1.0	1.0	1.0	2.0
*E. coli* KCTC2593	1.0	2.0	2.0	2.0	2.0	2.0	2.0	2.0	2.0	4.0	4.0
*E.coli* KCTC2571	1.0	2.0	2.0	4.0	2.0	2.0	4.0	2.0	2.0	4.0	4.0
*P. aeruginosa* KCTC1637	2.0	4.0	4.0	4.0	4.0	4.0	8.0	1.0	1.0	1.0	4.0
*P. aeruginosa* KCTC2004	2.0	4.0	8.0	8.0	4.0	8.0	8.0	2.0	2.0	8.0	8.0
*P. aeruginosa* KCTC2513	2.0	8.0	8.0	16	4.0	8.0	8.0	2.0	2.0	8.0	8.0
*S. typhimurium* KCTC1926	2.0	8.0	16	16	4.0	8.0	8.0	1.0	1.0	2.0	8.0
*B. subtilis* KCTC3068	1.0	4.0	4.0	4.0	2.0	4.0	4.0	1.0	1.0	2.0	4.0
*B. subtilis* KCTC1021	2.0	4.0	8.0	16	2.0	4.0	4.0	4.0	4.0	8.0	8.0
*B. subtilis* KCTC1022	1.0	1.0	1.0	2.0	1.0	1.0	1.0	2.0	2.0	4.0	4.0
*S. epidermidis* KCTC1917	2.0	2.0	2.0	4.0	2.0	2.0	2.0	1.0	1.0	4.0	4.0
*S. aureus* KCTC1621	1.0	1.0	4.0	4.0	2.0	4.0	4.0	1.0	2.0	2.0	4.0
*S. aureus* KCTC1916	1.0	1.0	1.0	2.0	2.0	2.0	4.0	2.0	2.0	4.0	4.0
*S. aureus* KCTC3881	2.0	2.0	2.0	4.0	2.0	2.0	4.0	1.0	2.0	4.0	4.0
Average MIC (µM)	1.5	3.2	4.6	6.3	2.6	3.9	4.6	1.6	1.8	4.0	5.0
MHC^b^ (µM)	1.6	13	100	50	50	200	200	100	200	800	400
Relative selective index^c^ (MHC/average MIC)	1.1	4.1	22	8.0	19	51	43	63	111	200	80

a Minimum inhibitory concentrations(MICs)were determined in three independent experiments performed in triplicate with a standard deviation of 14.0%.

b The minimal peptide concentration that produced hemolysis. When no detectable hemolysis was observed at 100 µM, a value of 200 µM was used to calculate the therapeutic index.

c The ratio of the MHC (µM) over the averageMIC (µM). Larger values indicate greater cell selectivity.

**Table 3:** Antimicrobial activity of Pis-1 and its analogs against standard bacterial strains.

	MIC^a^ (µM)										
Drug-resistant bacterial strains	Pis-1	Pis-F1A	Pis-F2A	Pis-F6A	Pis-F1K	Pis-F2K	Pis-F6K	Pis-V10K	Pis-F1K/V10K	Pis-F2K/V10K	Pis-F6K/V10K
MRSA CCARM3089	8.0	8.0	16	32	8.0	16	16	8.0	4.0	8.0	8.0
MRSA CCARM 3090	8.0	8.0	16	32	8.0	16	16	8.0	4.0	8.0	8.0
MRSA CCARM 3108	4.0	8.0	16	16	8.0	16	16	4.0	4.0	8.0	8.0
MRSA CCARM 3114	4.0	8.0	16	16	8.0	16	16	4.0	8.0	8.0	8.0
MRSA CCARM 3126	4.0	8.0	16	16	8.0	16	16	4.0	4.0	8.0	8.0
MDRST CCARM 8003	4.0	8.0	32	32	4.0	16	16	8.0	8.0	16	16
MDRST CCARM 8007	4.0	8.0	16	16	4.0	16	16	4.0	8.0	8.0	8.0
MDRST CCARM 8009	4.0	8.0	16	16	4.0	16	16	4.0	8.0	8.0	8.0
MDREC CCARM 1229	4.0	8.0	16	32	8.0	16	16	4.0	4.0	8.0	8.0
MDREC CCARM 1238	4.0	8.0	16	32	8.0	16	16	4.0	4.0	8.0	8.0
MDRAB CCARM 12005	2.0	2.0	4.0	4.0	2.0	2.0	4.0	2.0	2.0	2.0	4.0
MDRAB CCARM 12035	2.0	2.0	4.0	4.0	2.0	2.0	4.0	2.0	2.0	2.0	4.0
MDRAB CCARM 12036	2.0	2.0	4.0	4.0	2.0	2.0	4.0	2.0	2.0	2.0	4.0
MDRAB CCARM 12037	2.0	2.0	4.0	4.0	2.0	2.0	4.0	2.0	2.0	2.0	4.0
MDRPA CCARM 2002	2.0	2.0	4.0	4.0	2.0	2.0	4.0	2.0	2.0	2.0	4.0
MDRPA CCARM 2003	2.0	2.0	4.0	4.0	2.0	2.0	4.0	2.0	2.0	2.0	4.0
MDRPA CCARM 2095	2.0	2.0	4.0	4.0	2.0	2.0	4.0	2.0	2.0	2.0	2.0
MDRPA CCARM 2163	2.0	2.0	4.0	4.0	2.0	2.0	4.0	2.0	2.0	2.0	2.0
Average MIC(µM)	3.6	5.3	12	15	4.7	9.8	11	3.8	4.0	5.8	6.4
MHC^b^(µM)	1.6	13	100	50	50	200	200	100	200	800	400
Relative selective index^c^(MHC/Average MIC)	0.44	2.5	8.3	3.3	11	20	18	26	50	138	63

a Minimum inhibitory concentrations (MICs) were determined in three independent experiments performed in triplicate with a standard deviation of 14.0%.

b The minimal peptide concentration that produced hemolysis. When no detectable hemolysis was observed at 100 µM, a value of 200 µM was used to calculate the therapeutic index.

c The ratio of the MHC (µM) over the average MIC (µM). Larger values indicate greater cell selectivity.

In an attempt to design a non-cytotoxic analog, we substituted Val^10^ in Pis-1 with Lys to produce Pis-V10K. This analog showed increased amphipathicity as well as decreased hydrophobicity compared to Pis-1. The sustained antibacterial activity of Pis-V10K was similar to that of Pis-1. We further substituted the Phe residues of Pis-V10K with Lys; these analogs showed 1.1–3.3-fold lower antibacterial activity than Pis-1 (Tables 3 and 4). Among the Pis-V10K analogs with further Lys substitution of Phe^1^, Phe^2^, and Phe^6^, Pis-F6K/V10K showed the lowest antibacterial activity. These data indicated that Phe^6^ is the most important for the antibacterial activity of Pis-V10K among three phenylalanines. We calculated the average of the MIC values of all bacterial strains for an overall evaluation of peptide-antimicrobial activity against both standard and drug-resistant bacterial strains. The antimicrobial activity of the V10K-series of peptides decreased in the following order: Pis-1≈Pis-V10K≈Pis-F1K/V10K> Pis-F2K/V10K> Pis-F6K/V10K.

**Table 4:** Percentage of α-helical Contents of the Peptides in 50 mM DPC and 100 mM SDS Micelles.

Peptide	100 mM SDS	50 mM DPC
Pis-1	94	94
Pis-V10K	96	100
Pis-F1K/V10K	93	95
Pis-F2K/V10K	88	78
Pis-F6K/V10K	90	94

### Cytotoxicity against Mammalian Cells

**Figure 2:**
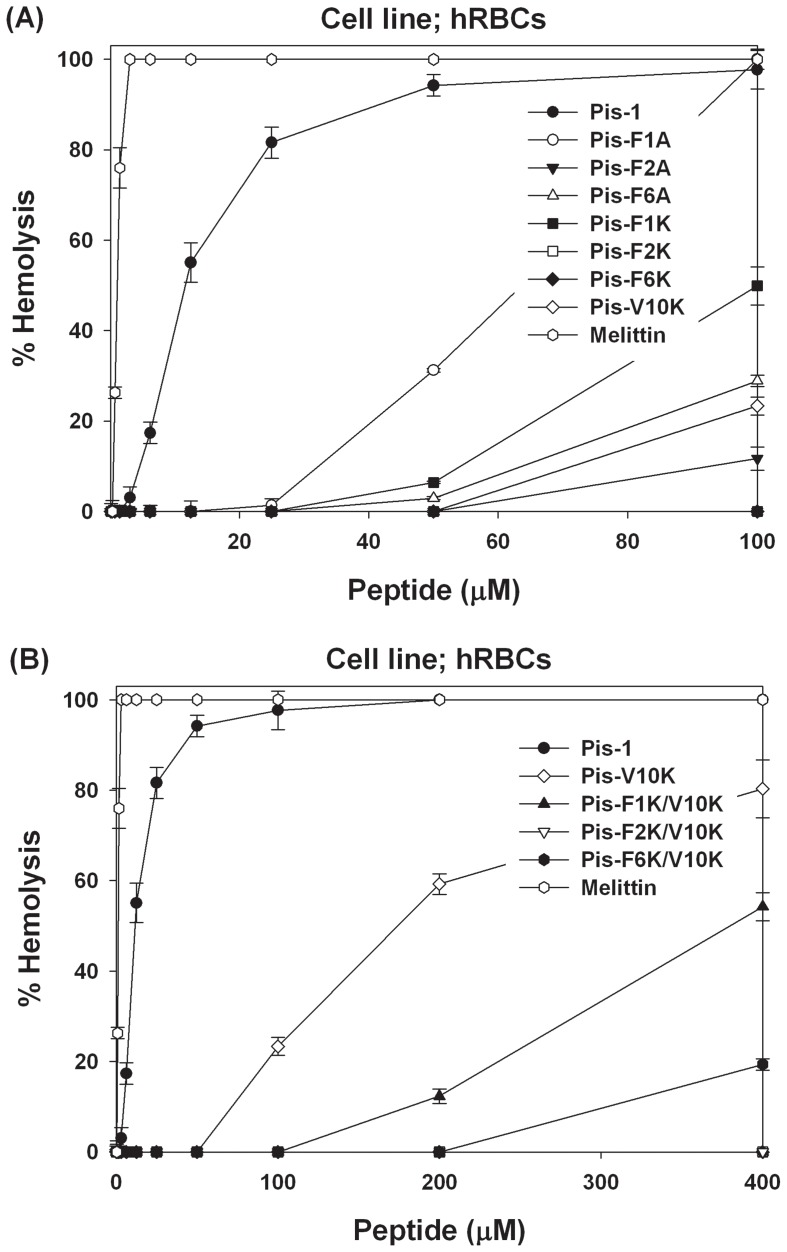
Dose-response curve for hemolytic activity of the peptides against human red blood cells (hRBCs). (A) Dose-response curve for hemolytic activity of peptide concentrations from 0 to 100 µM.

We next measured the cytotoxicity of the peptides against mammalian cells by measuring their abilities to lyse human erythrocytes. Dose-response curves of the hemolytic activities are shown in Fig. 2. Pis-1 analogs in which each Phe was substituted with Ala had stronger hemolytic activity than most peptide analogs in which Phe was substituted with Lys. At 100 µM, Pis-F1A produced 100% hemolysis, whereas Pis-F1K induced 50% hemolysis. Pis-F2K and Pis-F6K did not produce any hemolysis, whereas Pis-F2A and Pis-F6A produced>12% and 29% hemolysis, respectively, at concentrations of 100 µM. This result implies that of the three phenylalanine residues in Pis-1, Phe^2^ and Phe^6^ play important roles in Pis-1 cytotoxicity against hRBCs. Pis-1 was very cytotoxic and produced hemolysis even at its MIC, whereas Pis-V10K produced no hemolysis at 50 µM and only 20% hemolysis at 100 µM. The Lys^10^-substituted analogs, Pis-F1K/V10K, Pis-F2K/V10K, and Pis-F6K/V10K, exhibited no hemolysis, even at 100 µM. Pis-F1K/V10K induced 12% hemolysis at 200 µM and Pis-F6K/V10K induced 19% hemolysis at 400 µM, whereas Pis-F2K/V10K induced no hemolysis even at 400 µM (minimal hemolysis concentration (MHC) of 800 µM), as shown in Fig 2B. All three Lys^10^-substituted analogs showed high antibacterial activities with bacterial cell selectivities. Combined with the antibacterial activity data, these results show that Pis-F2K/V10K, in which the Phe^2^ residue had been replaced with Lys shows highest bacterial cell selectivities.

### Cytotoxicity against NIH3T3 Cell Lines

**Figure 3:**
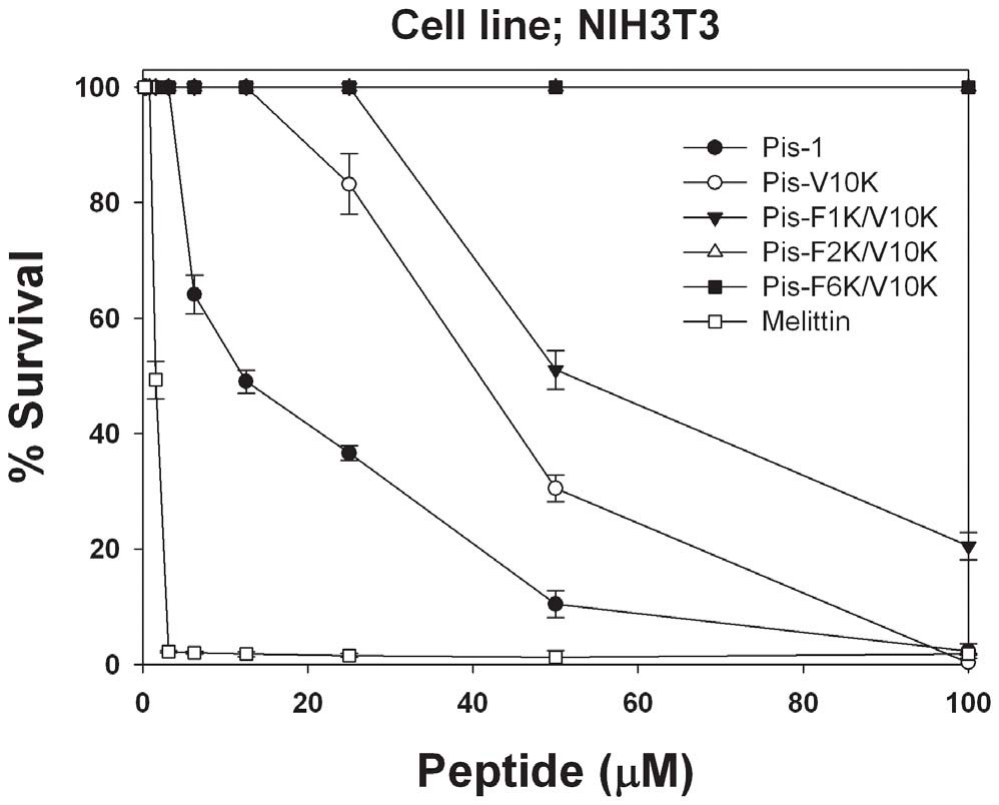
Dose-response curves for the cytotoxicity of peptides toward cells of the mouse embryonic fibroblast-derived NIH 3T3 cell line.

We examined the cytotoxicity of Pis-V10K-series peptides of low hemolytic activities toward NIH3T3 mammalian cells to investigate their potential therapeutic applications. As shown in Fig. 3, the survival rate of NIH3T3 cells decreased (64%, 49%, 37%, and 11%) with increasing concentration of Pis-1 (6.25, 12.5, 25, and 50 µM). Following treatment with Pis-V10K, survival rates were 100%, 83%, and 31% at 12.5, 25, and 50 µM, respectively. Administration of 25, 50, and 100 µM of Pis-F1K/V10K produced survival ratesof 100%, 51%, and 21%, respectively, whereas administration of both Pis-F2K/V10K and Pis-F6K/V10K produced a 100% survival rate, even at 100 µM. Therefore, hemolytic activity as well as this survival rate of NIH3T3 cells showed that Pis-F2K/V10K has the highest bacterial cell selectivity.

### CD Measurement of Pis-1 and Its Analogues

**Figure 4:**
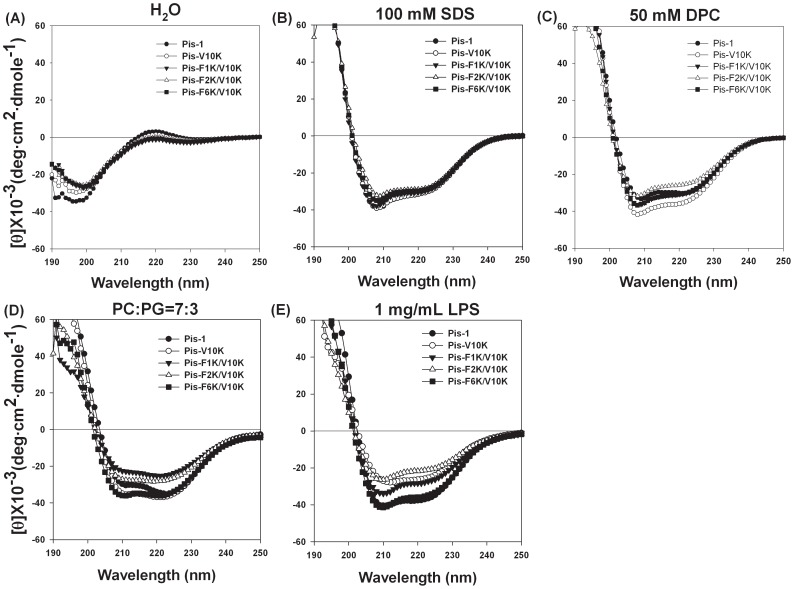
CD spectra of Pis-1 and its analogues in (A) H_2_O, (B) 50 mM DPC, (C) 100 mM SDS micelles, (D) 1 mM SUVs of PC:PG (7∶3, w/w), and (E) 1 mg/mL LPS solution.

We investigated the secondary structures of Pis-1 and its analogues in membrane-like environments by analyzing their CD spectra when dissolved in various kinds of membrane-mimetic environments. As shown in Fig.4, Pis-1 and its analogues had random structures in aqueous solution, but they showed conformational changes and formed α-helical conformations in 1 mg/mL LPS and 1 mM PC/PG SUVs as well as 50 mM DPC and 100 mM SDS micelles. In tested membrane mimetic environments, peptides exert double negative maxima at 205 nm and 220 nm, indicating α-helical structure. Helicity contents of the peptides in different membrane-mimetic environments were calculated as listed in Table 4. Pis-V10K with higher amphipathicity showed a slightly higher α-helicity than Pis-1. The other V10K series peptides containing fewer Phe residues than Pis-V10K showed a lower α-helicity than Pis-V10K, implying that the interaction between Phe and the lipid membrane might be important for the α-helicity of the peptide. Since each lipid exhibits specific electrostatic and hydrophobic interactions with peptides and aromatic residues, different membrane-mimetic environments with different lipids can cause different CD spectra of each analogs. We also performed NMR experiments to determine the tertiary structures of Pis-V10K. NOE connectivities of Pis-V10K were almost identical to those of Pis-1 and Pis-V10K has a very similar α-helical structure from Phe^2^ to Thr^21^ to that of Pis-1 (data not shown).

### Tryptophan Fluorescence Experiments

Phe and Trp residues present in many types of antibacterial peptides such as melittin, mastoparan B, IsCT, and piscidin have been reported to be critical for antibacterial or hemolytic activity [15], [25]–[27]. In mellitin, mastoparan, and IsCT, the Trp residues are located in the peptides’ hydrophobic core, showrestricted motion in the membrane, and are involved in the hydrophobic interactions with the acyl chains of phospholipids. These qualities are critical not only for antibacterial activity but also for cytotoxicity. Pis-1 possesses three Phe residues, at positions 1, 2, and 6, which play important roles in its antibacterial activity and cytotoxicity. To investigate the role of each Phe in the peptide’s interaction with phospholipid membranes, we synthesized Pis-F1W, Pis-F2W, and Pis-F6W, replacing one Phe each with Trp. To mimic the bacterial cell membrane, we utilized negatively charged SUVs composed of 7∶3 (w/w) EYPC/EYPG. We used neutral SUVs composed of 10∶1 (w/w) EYPC/CH SUVs to mimic mammalian cells. The fluorescence emission of the tryptophan residues of these peptides was then monitored in Tris-HCl buffer (pH 7.4) or in the presence of vesicles composed of either zwitterionic phospholipids [EYPC/CH (10∶1, w/w) SUVs] or negatively charged phospholipids (Table 5, Fig. S1). Melittin, which is known to exhibit not only high antibacterial activity but also high cytotoxicity, showed large blue shifts in both vesicles, implying that melittin is not cell selective. Pis-F1W exhibited a smaller blue shift in both vesicles compared to other peptides, indicating that the Phe^1^ residue inserted itself shallowly into both the zwitterionic phospholipid vesicles (mimicking mammalian membranes) and the negatively charged vesicles (mimicking bacterial membranes). This result implied that the Phe^1^ residue at the N-terminus does not play a critical role in cytotoxicity or antibacterial activity. In contrast, Pis-F2W exhibited the largest blue shifts of any peptide in zwitterionic phospholipid vesicles, suggesting that Phe^2^ is deeply inserted into the hydrophobic environment of the membrane interior, and that it plays a critical role in the antibacterial activity and cytotoxicity of Pis-1. Pis-F6W also exhibited a larger blue shift in the negatively charged vesicles compared to the zwitterionic phospholipid vesicles, indicating that the Phe^6^ of Pis-1 was more deeply inserted into the bacterial-mimetic vesicles than into the mammalian-mimic vesicles and plays a considerable role in its antibacterial activity.

**Table 5:** Tryptophan emission maxima of the peptides in Tris-HCl buffer (pH 7.4) or in the presence of EYPC/EYPG (7∶3, w/w) liposomesand EYPC/cholesterol (10∶1, w/w) liposomes.

Peptides	Sequences	Tris-HCl buffer (pH 7.4)	EYPC/EYPG (7∶3, w/w)	EYPC/CH (10∶1, w/w)
Pis-F1W	WFHHIFRGIVHVGKTIHRLVTG	352	340 (−12)	338 (−14)
Pis-F2W	FWHHIFRGIVHVGKTIHRLVTG	352	326 (−26)	330 (−22)
Pis-F6W	FFHHIWRGIVHVGKTIHRLVTG	352	332 (−24)	334 (−16)
Melittin	GIGAVLKVLTTGLPALISWIKRKREE-NH_2_	350	332 (−18)	332 (−18)

### Peptide-induced Permeabilization of Lipid Vesicles

**Figure 5:**
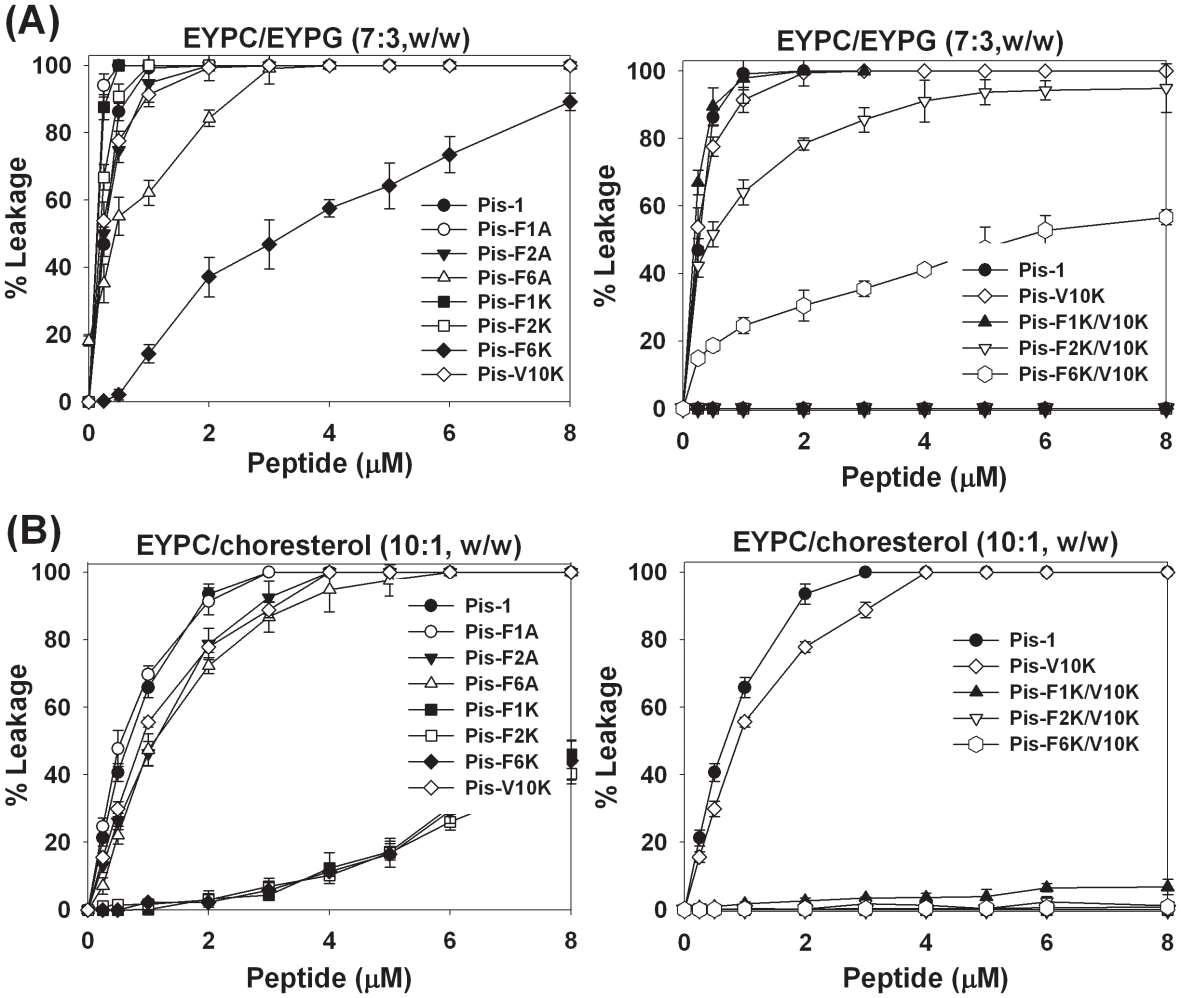
Peptide-induced permeabilization of lipid vesicles. Dose-response curves for calcein leakage from EYPC/EYPG (7∶3, w/w) LUVs (A), and EYPC/cholesterol (10∶1, w/w) LUVs (B) induced by the peptides.

To investigate the membrane-permeabilizing ability of the peptides, we measured the release of the fluorescent marker calcein from liposomes of various compositions, as described in the previous section. The percentage of calcein leakage, measured 3 min after exposure to each peptide, was used to assess the peptides’ ability to permeabilize the membrane. Fig. 5 shows the dose-response curve for peptide-induced calcein release. Peptides with substitution of Phe with Ala permeabilized the negatively charged LUVs (EYPC/EYPG (7∶3, w/w) more effectively than peptides with a substitution of Phe with Lys. As shown in Fig. 5A and 5B, peptides with a substitution of Phe^6^ with Ala or Lys induced much less calcein leakage than corresponding analogs with substitution of Phe^1^ or Phe^2^ with Ala or Lys in both types of negatively charged vesicles. Furthermore, Pis-F1K/V10K and F2K/V10K permeabilized negatively charged bacterial membranes much more effectively. Pis-F6K/V10K induced much less calcein dye leakage than Pis-F1K/V10K and Pis-F2K/V10K in negatively charged membrane. These results imply that the Phe^6^ residue of Pis-1 is much more effective in causing calcein dye leakagein negatively charged membranes (mimicking bacterial membranes) than the Phe^1^ and Phe^2^ residues, and that Phe^6^ is crucial for antibacterial activity. These results are concordant with the result showing that the MIC of Pis-F6K/V10K against 7 different standard strains was less active than the MICs of Pis-F1K/V10K and Pis-F2K/V10K. Therefore, the Phe^6^ residue of Pis-1 should be retained in development of novel antimicrobial peptides. As shown in Fig. 5C (left panel), the 8 µM concentration of Pis-F1K, Pis-F2K, and Pis-F6K produced approximately 40% calcein leakage in neutral vesicles, whereas the same concentration of Pis-1 and Pis-V10K induced 100% calcein leakage. As shown in Fig. 5D (right panel), at the 8 µM concentration, Pis-F1K/V10K induced 7% calcein release whereas Pis-F2K/V10K and Pis-F6K/V10K did not induce any calcein leakage in neutral vesicles. Collectively, these data show that Pis-F2K/V10K induced strong calcein leakage in negatively charged vesiclesbut did not induce calcein leakage in zwitterionic vesicles. Our results indicate that the relative abilities of the peptides to induce calcein leakage from negatively charged vesicles are concordant with their antibacterial activities. In addition, the data on production of calcein leakage from zwitterionic vesicles are concordant with the analogs’ relative hemolytic activities. Based in its antibacterial activity, hemolytic activity, and fluorescence data, we can conclude that Pis-F2K/V10K, in which the Phe^2^ is substituted with Lys, shows the highest antibacterial activity and the lowest hemolytic activity among the three analogs with Phe substitutions.

### Inhibition of NO Production in LPS-stimulated RAW264.7 Cells

**Figure 6:**
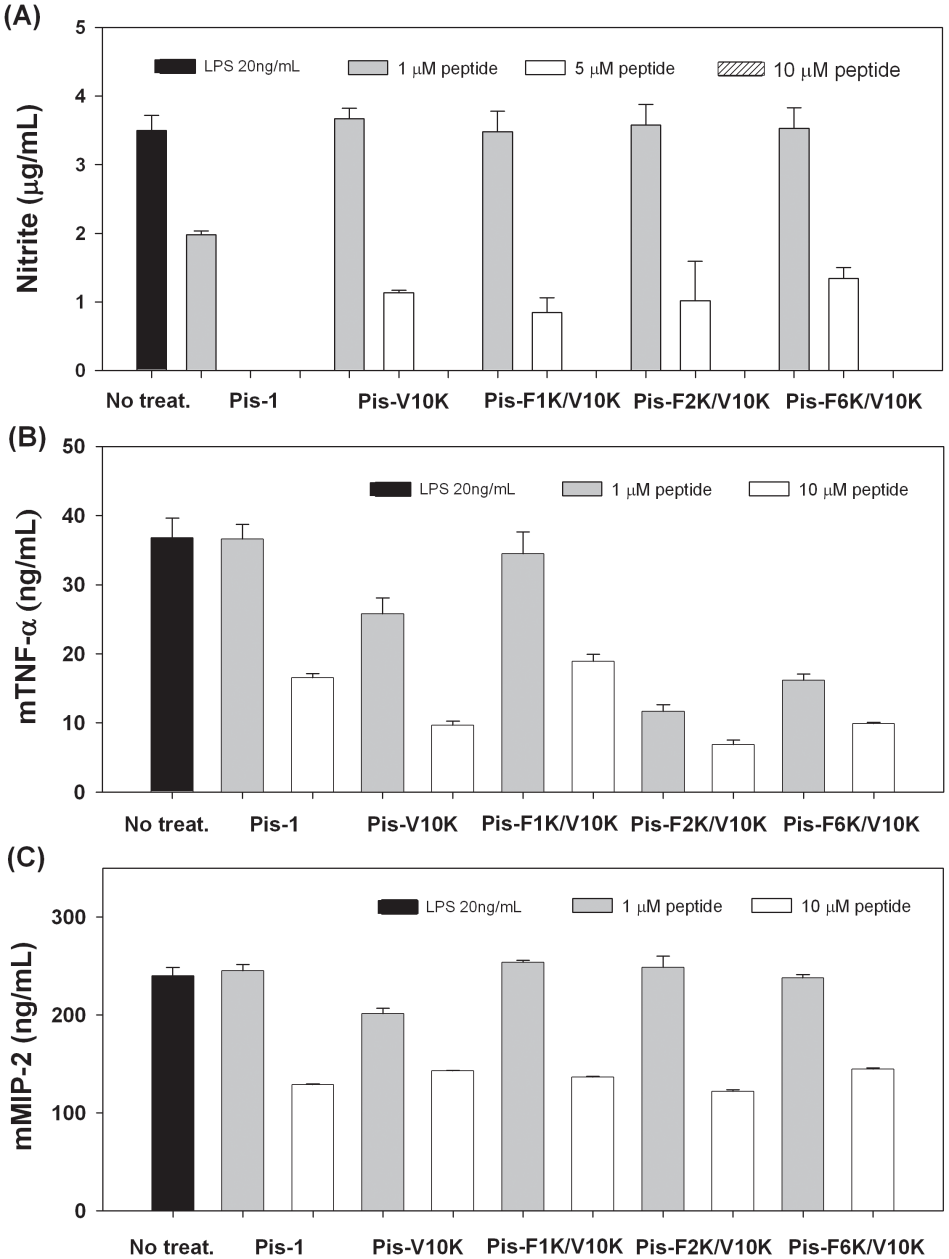
Inhibition of NO and cytokine production by Pis-1, Pis-V10K, Pis-F1K/V10K, Pis-F2K/V10K, and Pis-F6K/V10K in LPS-stimulated RAW264.7 cells. (A) Inhibition of NO production in LPS-stimulated RAW264.7 cells, (B) Inhibition of mTNF-α production in LPS-stimulated RAW264.7 cells, and (C) Inhibition of mMIP-2 production in LPS-stimulated RAW264.7 cells.

Pis-1 was strongly cytotoxic and produced hemolysis even at its MIC, whereas the Pis-V10K and Pis-V10K analogs produced no hemolysis at 50 µM. For this reason, Pis-V10K and its Pis-V10K analogs were used in further study to design potent peptide antibiotics. To investigate the anti-inflammatory activity of Pis-1 analogs, we directly measured the peptide-mediated inhibition of NO production in LPS-stimulated RAW264.7 cells, using peptide concentrations of 1, 5, and 10 µM. As shown in Fig. 6A, concentrations of 1–10 µM of Pis-1, Pis-V10K, Pis-F1K/V10K, Pis-F2K/V10K, and Pis-F6K/V10K gradually inhibited NO production in LPS-stimulated RAW264.7 cells. At both 5 µM and 10 µM, Pis-1 inhibited NO production completely; at these levels, Pis-1 also caused hemolysis. Concentrations of 5 µM Pis-V10K, Pis-F1K/V10K, Pis-F2K/V10K, and Pis-F6K/V10K inhibited NO production by 68%, 76%, 71%, and 62%, respectively, compared to NO production in untreated cells. All Pis-V10K-series peptides exhibited anti-inflammatory activityat concentrations up to 5 µM while this concentration was not hemolytic.

### Quantification of Inflammatory Cytokines (mTNF-α and mMIP-2)

We directly investigated mRNA expression of inflammation-related cytokines (mTNF-α and mMIP-2) in LPS-stimulated RAW264.7 macrophages performing quantitative analysis of mTNF-α and mMIP-2. TNF-α is known to be a key regulator of inflammation and defense mechanisms against bacterial infection; MIP-2 induces the release of other pro-inflammatory cytokines such as interleukin(IL)-1, IL-6, and TNF-α and is crucial for producing immune responses against inflammation or infection. As shown in Fig. 6B and 6C, administration of the peptides at 1–10 µM gradually suppressed mTNF-α and mMIP-2 production in LPS-stimulated macrophages. Pis-1 was strongly cytotoxic and produced hemolytic activity even at its MIC, whereas all Pis-V10K-series peptides showed high bacterial cell selectivity at 10 µM. These experiments demonstrated that both mTNF-α and mMIP-2 production were most strongly suppressed (82% and 50%, respectively, compared to untreated cells) in Pis-F2K/V10K-treated (10 µM) macrophages. In particular, significantly decreased expression of mTNF-α implies that Pis-F2K/V10K exhibits the highest bacterial cell selectivity among all the analogs, and shows potential as a potent peptide antibiotic. Therefore, Pis-F2K/V10K was used for further study to investigate anti-inflammatory activities and inflammatory response pathways.

### RT-PCR

**Figure 7:**
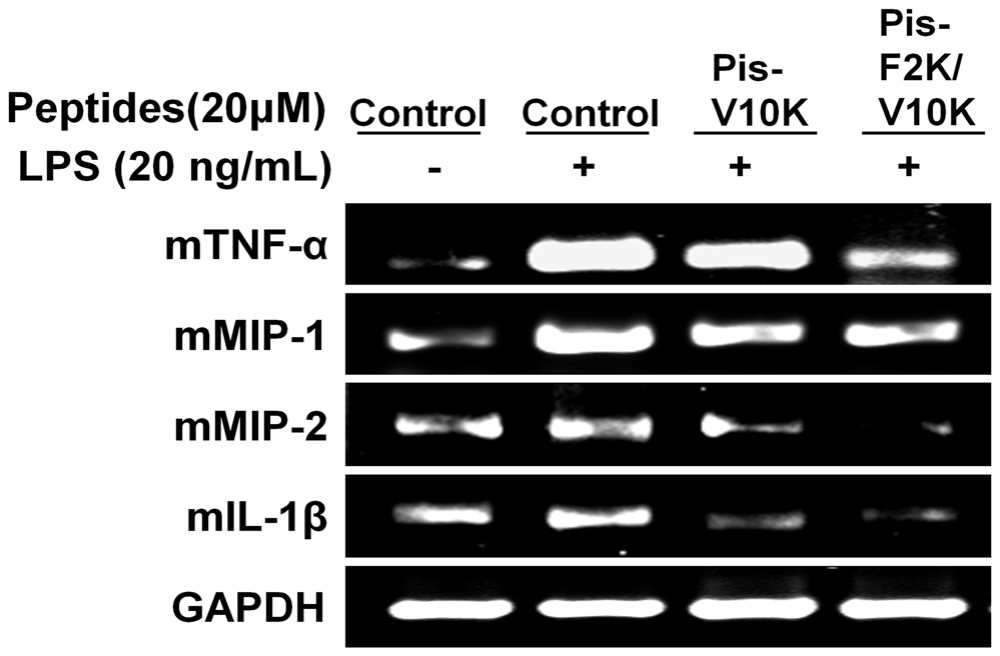
Reverse transcription-polymerase chain reaction. Inhibition of inflammatory cytokines (mTNF-α, mMIP-1, mMIP-2, and mIL-1β) by Pis-V10K and Pis-F2K/V10K in LPS-stimulated RAW264.7 cells.

We evaluated whether piscidin analogs suppresses LPS-dependent production of inflammatory-related cytokines (mTNF-α, mMIP-1, MIP-2, and mIL-1β). As shown in Fig. 7, LPS treatment caused a significant increase in cytokines (mTNF- α, mMIP-1, MIP-2, and mIL-1β) at 3 h after LPS challenge, whereas pre-treatment with Pis-V10K and Pis-F2K/V10K significantly decreased cytokine mIL-1β levels by 73.0% and 85.8%, respectively. The expression of mMIP-2 mRNA was inhibited by 54.4% and 94.3%, respectively, in Pis-V10K- and Pis-F2K/V10K-treated (20 µM) cells, compared to mRNA levels in untreated cells (Fig. 7). In summary, Pis-F2K/V10K suppressed the mRNA expression of all cytokines investigated more effectively than Pis-V10K.

### Western blot

**Figure 8:**
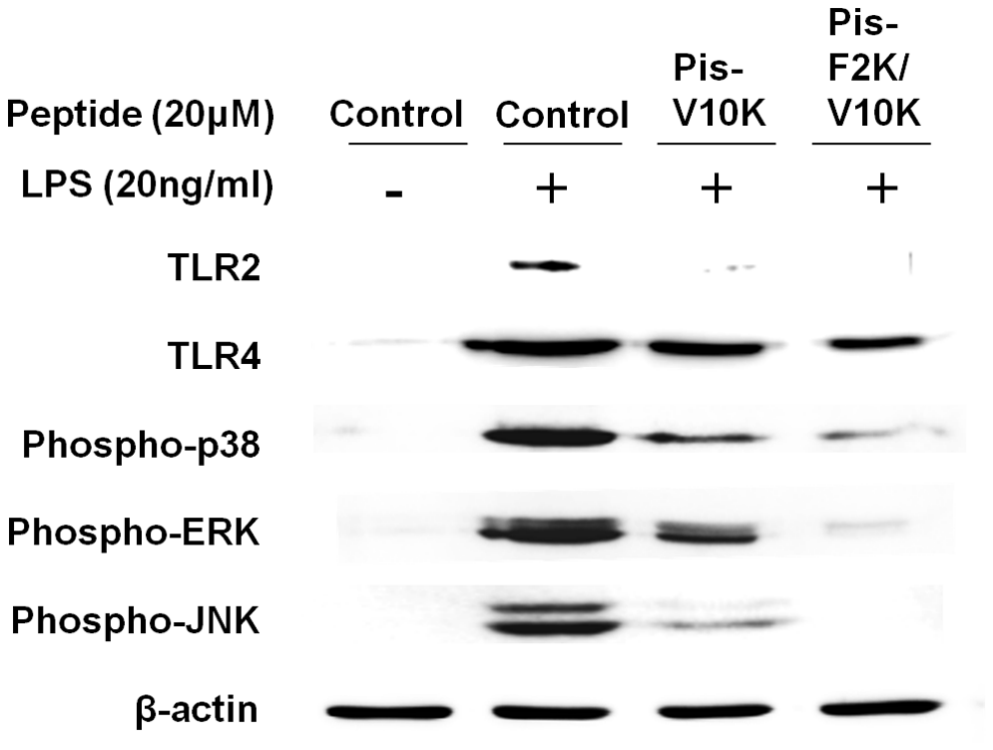
Anti-inflammatory activities of Pis-V10K and Pis-F2K/V10K in LPS-stimulated RAW264.7 cells. Effects of Pis-V10K and Pis-F2K/V10K on TLR2, TLR4, phospho-ERKs, phosphor-JNK, and phosphor-p38 MAPK. TLR2, TLR4, phospho-ERKs, phosphor-JNK, and phosphor-p38 MAPK protein levels were determined by western blot analysis using specific antibodies.

We next examined the inflammatory response pathways of Pis-V10K and Pis-F2K/V10K. Toll-like receptor (TLR) family members are transmembrane proteins that play a critical role in signal transduction. Activated TLRs recognize pathogenic molecules and recruit downstream signaling proteins, including myeloid differentiation primary response 88 (MyD88), toll-interleukin 1 receptor (TIR) domain-containing adaptor protein/MyD88 adapter-like (TIRAP/Mal), and TIR domain-containing adapter protein inducing IFN-β (TRIF). This leads to secretion of various inflammatory proteins, including NO, IL-1β, and TNF-α. Extracellular signal-regulated kinases (ERKs), p38 MAP Kinase (MAPK), and c-Jun N-terminal kinases (JNKs) are members of the MAP kinase family of signal transduction proteinsthat playa role in a signaling cascade that is activated by various extracellular signals, including inflammatory cytokines, LPS, growth factors and stress, and controls cellular responses to these stimuli. Therefore, we investigated the ability of the peptide to suppress TLR2 and TLR4 expression using western blot analysis of cytoplasmic extracts from LPS-stimulated RAW264.7 cells. As shown in Fig. 8, TLR2 and TLR4 expression as well as the increased phosphorylation of MAPKs were more effectively suppressed by treatment with 20 µM Pis-F2K/V10K than with Pis-V10K.

### Interaction of Peptides with FITC-labeled LPS Aggregates

**Figure 9:**
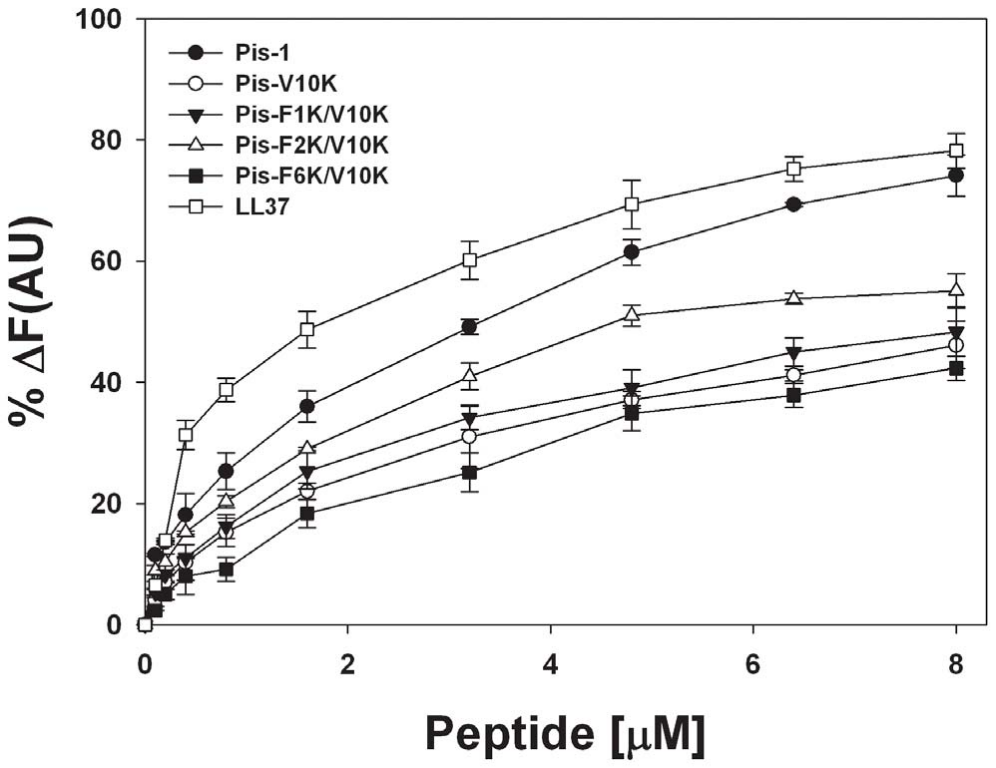
Enhancement of the intensity of FITC-labeled LPS as a function of Pis-1, Pis-V10K, Pis-F1K/V10K, Pis-F2K/V10K, Pis-F6K/V10K, and LL37 concentration. *AU*, absorbance unit.

The interaction between the antimicrobial peptides and LPS causes the dissociation of large LPS aggregates into smaller-sized ones, which can be demonstrated as increased fluorescence using FITC-conjugated LPS. In this study, we investigated the interaction of Pis-1, Pis-V10K, Pis-F1K/V10K, Pis-F2K/V10K, Pis-F6K/V10K, and LL37 with LPS. The cathelicidin LL37 is a human derived AMPs and is important effector molecule of innate immunity [28]. The changes in the intensity of FITC-LPS fluorescence implied that the addition of peptides induced the dissociation of LPS aggregates as the peptides interacted with FITC-LPS. As shown in Fig. 9, LL37 exhibited deep dequenching and the highest fluorescence intensity for FITC-labeled LPS among all peptides. Pis-1 showed less fluorescence intensity than LL37, but was significantly better at breaking up LPS aggregates than all Pis-1 analogs. Pis-F2K/V10K showed higher fluorescence intensity than Pis-F1K/V10K, Pis-V10K, or Pis-F6K/V10K, implying that Pis-F2K/V10K interacted with FITC-LPS more tightly, and induced the dissociation of LPS aggregates more effectively than other V10K-series peptides. These results indicates that both positively charged residues and hydrophobic Phe residues are important for the interactions between LPS and the peptides.

#### NMR studies of peptides bound to LPS

**Figure 10:**
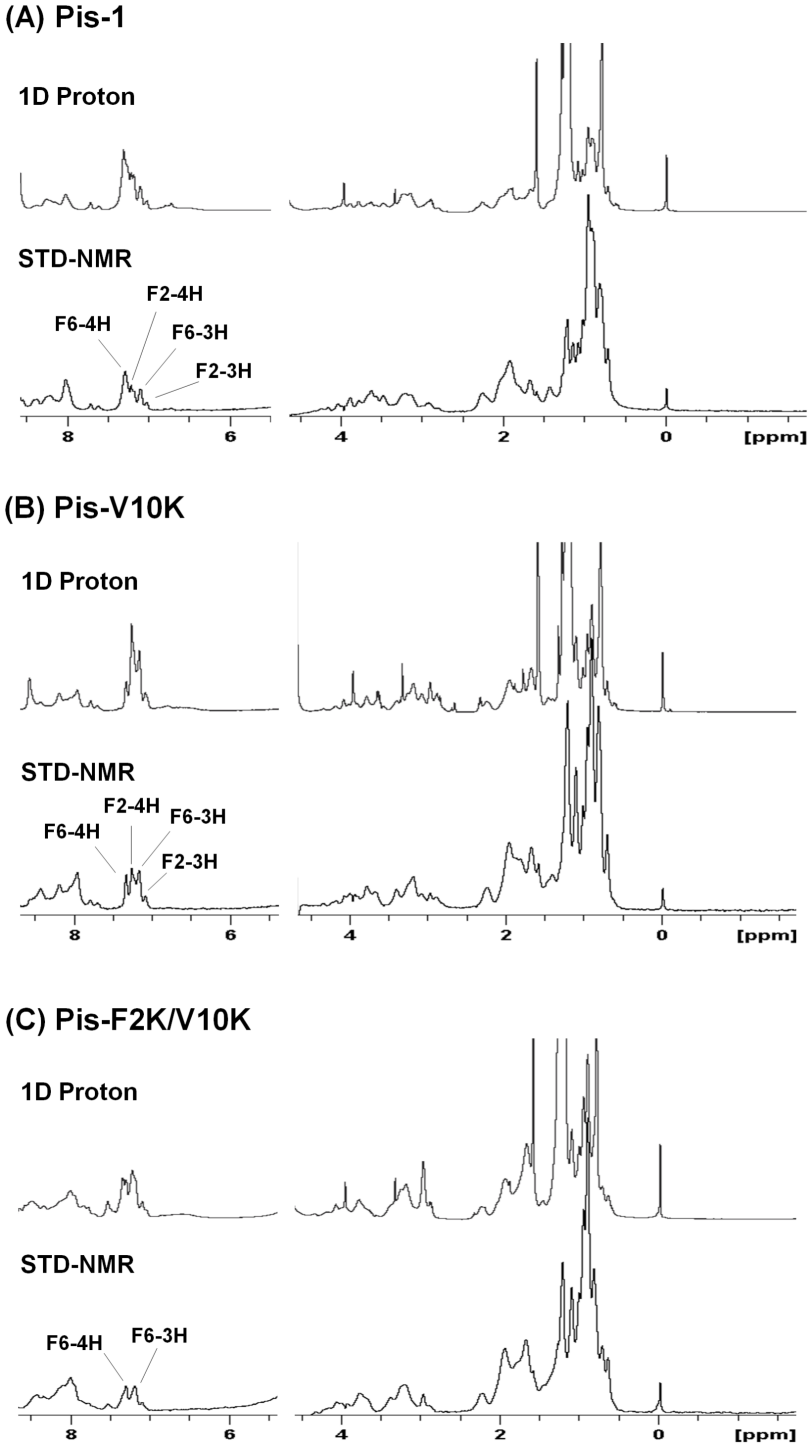
STD-NMR results showing the interaction between Pis-1 and its analogs with LPS. (A) Pis-1, (B) Pis-V10K, and (C) Pis-F2K/V10K. Spectral differences primarily constituted resonances belonging to peptide protons bound to LPS. STD experiments were carried out at a peptide concentration of 0.5 mM in presence of 0.1 mg/mL LPS in H_2_O, pH 5.9 at 298 K.

To understand the mode of peptideinteraction with LPS, we performed STD-NMR experiments. Fig.10 shows the one-dimensional spectra of free peptides and the STD spectra of piscidin analogs bound to LPS. STD spectra of Pis-1, Pis-V10K, and Pis-F2K/V10K show that thearomatic ring protons of Phes as well as a number of amide proton and aliphatic side-chain proton resonances are in close association with LPS. The aromatic ring protons of Phe^1^, Phe^2^ and Phe^6^ showed a strong STD effect in Pis-1 and Pis-V10K, andPis-F2K/V10K. These data indicated that they are intimately associated with LPS and Phes in the N-terminal helix of piscidin are in close contact with LPS or bacterial cell membrane.

## Conclusions

In this study, we designed Pis-1 analogs with substitution of the Val^10^ and Phe residues in an attempt to determine the roles of these residues, to develop novel peptide antibiotics with bacterial cell selectivity. We evaluated the antimicrobial activity, cytotoxicity against mammalian cells, interaction with phospholipid membranes, and anti-inflammatory activity of these analogs.

Compared to Pis-1, Pis-V10K with Lys^10^substitution showed sustained antibacterial activity and dramatically decreased cytotoxicity against mammalian cells. Pis-V10K did not produce hemolysis at concentrations <50 µM, whereas Pis-1 was highly cytotoxic against human erythrocytes even at its MIC. Therefore, Pis-V10K, which has a stable, linear, amphipathic α-helical structure, showed much higher bacterial cell selectivity than Pis-1. Both Pis-F2A and Pis-F2K showed much lower hemolytic activity than Pis-F1A or Pis-F6A and Pis-F1K or Pis-F6K, suggesting that Phe^2^strongly influences the cytotoxicity of Pis-1. Among Lys^10^-substituted peptides, Pis-F1K/V10K and Pis-F2K/V10K, (with Lys substitution of Phe^1^ or Phe^2^, respectively) showed higher antibacterial activity than Pis-F6K/V10K, suggesting that Phe^6^ plays a key role in the antibacterial activity of Pis-1 and should be retained in designed analogs. Pis-F1K/V10K, Pis-F2K/V10K, and Pis-F6K/V10K showed no hemolytic activity against hRBCs at 100 µM, and Pis-F2K/V10K did not show hemolytic activity even at 400 µM. Tables 2 and 3 list the MHC against hRBCs, the average MIC (AM) value for all bacterial strains, and the relative selectivity index (MHC/AM), which indicates the ability to effectively kill bacterial cells without exhibiting significant cytotoxicity toward mammalian cells. Thus, a high relative selectivity index is an indication of the two preferred characteristics of the peptide: a high MHC (low hemolytic activity) and a low MIC (high antimicrobial activity). Pis-1 had the lowest relative selectivity index (RSI; 1.1 against standard bacterial strains and 0.44 against drug-resistant bacterial strains). Pis-F2K/V10K had the highest RSI (200 against standard bacterial strains and 138 against drug-resistant bacterial strains), whereas all other Lys^10^-substituted analogs had lower RSI values than Pis-F2K/V10K, ranging from 50 to 111. This result implied that the Phe^2^ residue of Pis-1 plays a critical role in cytotoxicity against human red blood cells. Therefore, it is evident that retention of Phe^6^ and substitution of Phe^2^ with Lys as well as of Val^10^ with Lys improved bacterial cell selectivity. Collectively, these results imply that Pis-F2K/V10K shows potential as a candidate for development of potent antibiotics with novel antibacterial activity without mammalian cytotoxicity.

We then investigated the mechanism of action. Most bacterial cell-selective antimicrobial peptides bind strongly and permeate more efficiently into negatively charged phospholipid membranes, which mimic bacterial membranes, than into zwitterionic phospholipid membranes, which mimic the major component of the outer leaflet of human erythrocytes [29], [30]. To investigate the antimicrobial mechanism of action of the Pis-1 analogs, we compared their ability to damage mammalian- and microbial-mimetic membranes. Experiments employing a fluorescent dye entrapped within LUVs of varying phospholipid composition showed that all Pis-V10K-series peptides (at their MIC) induced very strong leakage of dye from negatively charged, bacterial cell membrane-mimetic phospholipid vesicles, but induced much less leakage of dye from neutral vesicles, which mimic mammalian cell membranes. Among all the analogs, Pis-F2K/V10K showed the strongest bacterial cell mimic selectivity. These results implied that the bactericidal action of all Pis-1 analogs is attributable to perturbation of the bacterial cell membrane.

Understanding the structural characteristics of peptides that allow for potent and selective activity against microbial cells would help in designing peptides for future therapeutic use. Recently, the high-resolution structure of Pis-1 has been investigated in various environements using solid-state NMR, solution NMR spectroscopy as well as MD simulation [13], [14], [31]–[35]. Pis-1 has an α-helix from Gly^8^ to His^17^ in dodecylphosphocholine (DPC) micelles, which mimic a neutral membrane system [31]. However, in SDS micelles, which mimic a negatively charged bacterial cell membrane, Pis-1 has an α-helical structure from Phe^2^ to Gly^22^ [13]. Because piscidin is a highly positively charged amphipathic peptide, it is possible that it adopts a more stable α-helical conformation in negatively charged SDS micelles than in neutral DPC micelles. Gly^8^ is located at the boundary of the hydrophobic and hydrophilic phase of the amphipathic α-helix of Pis-1. Gly is known to provide big flexibility in structure of protein or peptide because it does not have bulky side chain. Furthermore, it has been reported that solid-state structures in 3∶1 PC/PG, which most closely resemble the 7∶3 EYPC/PG vesicle environment, have a kink at Gly^13^ whereas Pis-1 has a kink at Gly^8^ in DPC micelles [31], [34]. Structural refinement with ^1^H-^15^N dipolar couplings and ^15^N chemical shifts measured by oriented sample solid-state NMR and MD simulations revealed a slight kink at Gly^13^ of Pis-1. This could lead to an optimization of its hydrophobic moment and making hydrophobic side chains interact optimally with the bilayer. Therefore, it is possible that the structure of Pis-1 can be a straignt helix or can be kinked at either Gly^8^ or Gly^13^, depending on different membrane environments [13], [31], [34]. Pis-1[PG], into which Pro has been introduced at position 8, shows sustained antimicrobial activity with bacterial cell selectivity. In Pis-1[PG], the N-terminal region is disrupted but the C-terminal amphipathic α-helix is retained in SDS micelles [13].

In this study, we found that Phe^2^ of Pis-1, plays a key role by enabling deeper insertion of Pis-1 into bacterial-mimic membranes, increasing its anchoring potential. Trp blue shift and calcein dye leakage studies revealed that the Phe^2^ residue near the N-terminus of Pis-1 deeply interted into the membrane and showed strong interaction with mammalian as well as bacterial-mimetic membranes. Accordingly, it can be concluded that the penetration of Phe^2^ into the membrane environment contributes to the toxicity of Pis-1, as well to its high antibacterial activity. We also investigate the anti-inflammatory activities of Pis-1 and its V10K analogs. All Lys^10^-substituted peptides showed anti-inflammatory effects as well as potent antibacterial activities against all bacterial strains, withstrong bacterial cell selectivities. Among all peptides tested, the Pis-F2K/V10K peptide, with substitutions of Phe^2^ and Val^10^ with Lys, showed the highest anti-inflammatory activity, suppressing NO production and inflammatory related cytokines, as well as the expression of closely related inflammation-related cytokines such as mTNF-α, mMIP-1, mMIP-2, and mIL-1β. Western blot analysis indicated that Pis-V10K and Pis-F2K/V10K may disrupt binding of LPS to TLRs and eventually suppress the MAPKs signaling pathways in LPS-stimulated macrophage-derived RAW 264.7 cells. We also investigated the interactions between V10K-series peptides and LPS using fluorescence-labeled LPS. Pis-F2K/V10K caused greater dissociation of the aggregated LPS than other V10K-series peptides, implying effective interactions between Pis-F2K/V10K and LPS. Among the Pis-V10K-series peptides, Pis-F2K/V10K and Pis-F6K/V10K showed the lowest cytotoxicity toward mouse embryonic fibroblast-derived NIH3T3 cell lines.

In conclusion, Phe^2^ plays key roles in the cytotoxicity as well as the antibacterial activities of Pis-1 whereas Phe^6^ is essential for its antibacterial activities. Pis-F1K/V10K, Pis-F2K/V10K, and Pis-F6K/V10K showed no hemolytic activity against hRBCs at 100 µM and have braod spectrum of antibacterial and anti-inflammatory activities. Collectively, our resultsshow that Pis-F2K/V10K has highest bacterial cell selectivity among all analogs. We derived potent antibacterial and anti-inflammatory agents with no toxicity toward mammalian cells from Pis-1, which they are strong candidates for potent peptide antibiotics suitable for treating the endotoxic shock caused by bacterial infections. Further study will be required to investigate the mechanism underlying the anti-inflammatory activities of these peptides.

## Supporting Information

Figure S1 **Tryptophan emission maxima of the peptides in Tris-HCl buffer (pH 7.4) or in the presence of EYPC/EYPG (7∶3, w/w) liposomesand EYPC/cholesterol (10∶1, w/w) liposomes.**
(TIF)

Table S1 **Antimicrobial activity of Pis-1 and its analogs including Trp-containing mutants against standard bacterial strains.**
(DOCX)
