# Supplementary material for: Role of Phenylalanine and Valine10 Residues in the Antimicrobial Activity and Cytotoxicity of Piscidin-1
Source: PLoS One. 2014 Dec 4;9(12):e114453. doi: 10.1371/journal.pone.0114453 (PMC4256409; doi:10.1371/journal.pone.0114453)
Supplement: Table S1 — Antimicrobial activity of Pis-1 and its analogs including Trp-containing mutants against standard bacterial strains. (DOCX) [file pone.0114453.s002.docx]

**Table S1. Antimicrobial activity of Pis-1 and its analogs including Trp-containing mutants against standard bacterial strains.**


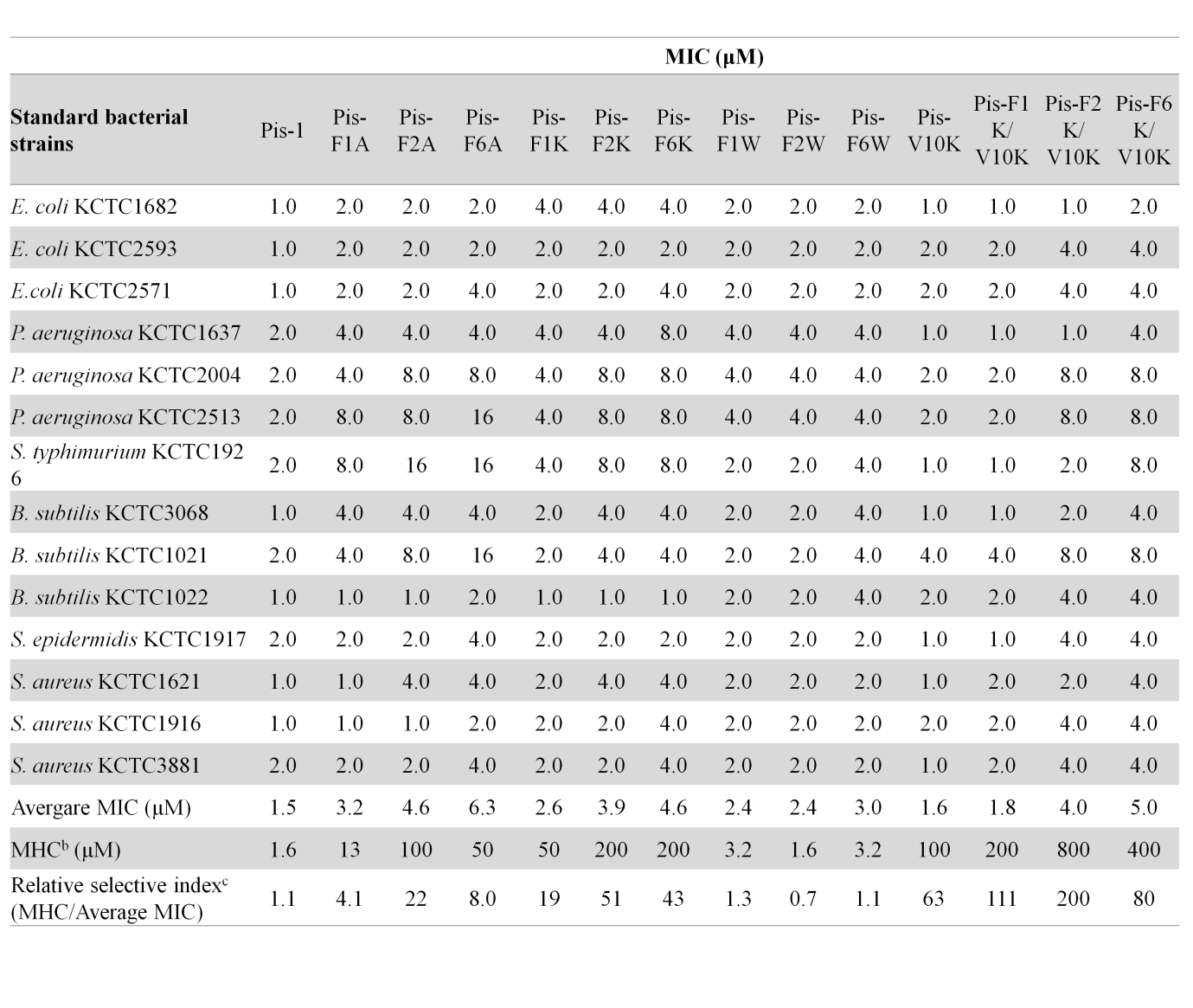


aMinimum inhibitory concentrations(MICs)were determined in three independent experiments performed in triplicate with a standard deviation of 14.0%.

bThe minimal peptide concentration that produced hemolysis. When no detectable hemolysis was observed at 100 μM, a value of 200 μM was used to calculate the therapeutic index.

cThe ratio of the MHC (μM) over the averageMIC (μM). Larger values indicate greater cell selectivity.
